# Mean Platelet Volume and Its Association With In-Hospital Outcomes in Patients With Acute ST-Segment Elevation Myocardial Infarction Undergoing Primary Percutaneous Coronary Intervention

**DOI:** 10.7759/cureus.55606

**Published:** 2024-03-05

**Authors:** Özlem Özen Ekmekci, Gürkan Karaca, Ali Kimiaei, Seyedehtina Safaei, Ahmet Ekmekci

**Affiliations:** 1 Family Medicine, Memorial Ataşehir Hospital, Istanbul, TUR; 2 Cardiology, Bahçeşehir University, Istanbul, TUR; 3 Medicine, Bahçeşehir University, Istanbul, TUR

**Keywords:** prognostic factors, cardiovascular disease, percutaneous coronary intervention, acute coronary syndrome, mean platelet volume

## Abstract

Background

Mean platelet volume (MPV), reflecting platelet size and activation, has been associated with cardiovascular disease (CVD) risk and mortality. Yet, its prognostic significance in acute coronary syndrome (ACS) patients undergoing primary percutaneous coronary intervention (PCI) remains uncertain. This study investigates whether elevated MPV levels upon admission in ST-segment elevation myocardial infarction (STEMI) patients predict adverse in-hospital outcomes after primary PCI.

Objectives

The aim of this study was to measure MPV in patients with STEMI who underwent primary PCI and to evaluate its association with in-hospital outcomes such as death, recurrent myocardial infarction, heart failure, and bleeding.

Methods

We enrolled 400 consecutive patients with STEMI (mean age 56.20 years, 356 males, 44 females) who underwent primary PCI at our center. We obtained MPV values from complete blood count tests performed at admission. We divided the patients into two groups based on the normal MPV range of 7.40 to 12 fL. We compared the baseline characteristics and in-hospital outcomes of the two groups. We used Cox proportional hazards regression analysis to adjust for potential confounders and evaluate the impact of MPV on in-hospital outcomes.

Results

There was no significant difference in MPV values between the two groups (9.10 ± 1.20 fL vs. 9.00 ± 1.10 fL, p = 0.54). Patients who died exhibited higher age, male predominance, hypertension, diabetes, a lower left ventricular ejection fraction, lower levels of low-density lipoprotein cholesterol, and lower levels of hemoglobin and hematocrit compared to survivors. MPV was not associated with any of the in-hospital outcomes in the unadjusted or adjusted analyses.

Conclusion

In this cohort of patients with STEMI who underwent primary PCI, admission MPV was not a predictor of in-hospital outcomes. Further studies are needed to clarify the role of MPV in the pathophysiology and prognosis of ACS.

## Introduction

Coronary artery disease (CAD) is a major global health problem that causes high mortality and morbidity [1]. The main pathophysiological mechanism of CAD and its complications is atherosclerosis, a chronic inflammatory process that affects the arterial wall [2]. Atherosclerosis develops over decades and leads to organ damage when it becomes clinically manifest [2].

The treatment of CAD depends on the stage and severity of the disease and may include palliative or specific therapies. Prevention of atherosclerosis and its risk factors is essential for improving the quality of life and survival of patients with CAD [3]. Management of CAD involves both primary and secondary prevention strategies [3,4].

Primary prevention aims to prevent or delay the onset of CAD by modifying the risk factors that predispose to atherosclerosis [3]. These risk factors include smoking, an unhealthy diet, obesity, physical inactivity, hypertension, dyslipidemia, diabetes, and genetic factors [3]. Primary prevention targets not only patients with CAD but also the general population at risk of developing the disease [3]. The promotion of a healthy lifestyle is the cornerstone of primary prevention and should be implemented at an early age [3]. Primary prevention also requires the regular screening and follow-up of high-risk individuals by physicians and the appropriate pharmacological intervention when indicated [3].

Secondary prevention aims to prevent or reduce the recurrence and severity of CAD events and complications in patients who have already been diagnosed with the disease [4]. Secondary prevention involves the control of the atherosclerotic process in the coronary arteries and the prevention of thrombotic complications that may result from plaque rupture or erosion [4]. Secondary prevention reduces the risk of non-fatal ischemic events and cardiac death [4]. Secondary prevention includes both lifestyle modification and pharmacological therapy, as well as surgical or interventional procedures when necessary [4].

Despite the advances in the identification and management of the classical risk factors for CAD, there is still a significant proportion of patients who develop the disease or experience adverse outcomes without having these risk factors [5]. For instance, about half of patients who present with acute myocardial infarction (AMI) or unstable angina pectoris (USAP) do not have any of the classical cardiovascular risk factors [5]. This suggests that there are other factors that contribute to the pathogenesis and prognosis of CAD that are not yet fully understood. Therefore, there is a need for further research on novel risk factors that may improve risk stratification and the prevention of CAD.

Atherogenesis, the formation of atherosclerotic plaques in the arterial wall, is a complex process that involves the interaction of various cellular and molecular components, such as platelets, white blood cells, endothelial cells, smooth muscle cells, and macrophages [6]. Atherosclerosis is the main cause of CAD, which can lead to acute ischemic syndromes, such as USAP, AMI, and sudden cardiac death [2]. These syndromes are triggered by the rupture or ulceration of the atherosclerotic plaque, which exposes the thrombogenic material to blood flow and activates platelets [6,7].

Platelets are essential for hemostasis, the process of stopping bleeding from injured vessels. Platelets adhere to the injured vessel wall and aggregate with each other to form a primary hemostatic plug, which is then stabilized by the activation of the coagulation cascade and the formation of a fibrin network [6]. Platelets are not uniform in size, density, and reaction [6]. Larger and more reactive platelets have higher hemostatic activity and are more prone to causing thrombosis [8]. Mean platelet volume (MPV) is a measure of the average size of platelets in circulation and reflects their reactivity. Elevated MPV is a potential risk factor for AMI, as it indicates the presence of more thrombogenic platelets [6]. Several studies have shown that MPV is higher in patients with acute coronary syndrome (ACS) than in those with stable angina pectoris (SAP) or healthy controls [9]. This suggests that MPV may reflect the severity and instability of the atherosclerotic plaque and the propensity for thrombus formation and occlusion of the coronary arteries [9]. Moreover, MPV has been found to be associated with the extent of myocardial damage and the prognosis of patients with AMI [10,11].

The mechanisms underlying the increase in MPV in CAD and AMI are not fully elucidated, but they may involve the stimulation of megakaryopoiesis, the production of platelets from their progenitor cells, and megakaryocytes in the bone marrow [12]. Megakaryocyte size and platelet size are positively correlated, and post-AMI bone marrow biopsies have shown an increase in the average cytoplasmic volume of megakaryocytes [12,13]. The study aims to assess whether high MPV levels at admission in patients with acute ST-segment elevation myocardial infarction (STEMI) predict worse in-hospital outcomes after primary PCI.

## Materials and methods

Study population

A retrospective evaluation was conducted on 400 consecutive STEMI patients admitted to the emergency department and undergoing emergency cardiac catheterization in the catheterization laboratory. Patients meeting the following criteria were enrolled in the study: symptom onset within 12 hours (or 18 hours for cardiogenic shock) of admission; chest pain lasting more than 30 minutes; ST-segment elevation of at least 2 mm in two or more contiguous electrocardiogram (ECG) leads or new-onset complete left bundle branch block; patients undergoing primary percutaneous coronary intervention (PCI with angioplasty and/or stent placement). The exclusion criteria were severe liver, kidney, or heart failure; use of oral anticoagulants; myeloproliferative disorders or anemia; pregnancy; and ACS other than STEMI.

Data collection and analysis of patient information

We collected data on clinical risk factors from previous medical records, including age, gender, diabetes mellitus (DM), hypertension, hypercholesterolemia, smoking, family history of cardiovascular disease (CVD), history of MI, and history of PCI or bypass surgery. We also recorded the time from symptom onset to angiography-reperfusion and the door-to-balloon time. We obtained blood values from medical reports before catheterization procedures. We measured MPV as part of an automated complete blood count using the Coulter LH 780 Hematology Analyzer (Beckman Coulter Ireland, Inc., Mervue, Galway, Ireland). We performed a 12-lead ECG for each patient immediately after admission and documented the MI type from the ECG. We obtained echocardiographic data from patient records and calculated the left ventricular ejection fraction (LVEF) using the modified Simpson’s method.

Coronary angiography, primary angioplasty, and stent implantation procedures

We administered chewable aspirin tablets (300 mg, unless contraindicated) and oral clopidogrel (300 mg loading dose) to all patients before coronary angiography. We evaluated the angiographic data of the patients and determined coronary anatomy. We administered heparin (10,000 U) to the patients and considered coronary artery stenosis of more than 50% as clinically significant. After angioplasty, we admitted all patients to the coronary intensive care unit and continued intravenous heparin at a rate of 500 U/hour or subcutaneous low-molecular-weight heparin at a dose of 1 mg/kg/day; all patients also continued with 100 mg aspirin and 75 mg clopidogrel. We used tirofiban at the discretion of the performing physician.

Definitions

Reperfusion time is the elapsed time from the onset of chest pain to the first balloon inflation. Door-to-balloon time is the time interval from hospital admission to balloon inflation.

Anemia during hospitalization is a hemoglobin concentration below 13 mg/dL in males and below 12 mg/dL in females. Cardiogenic shock is a state of significant and persistent hypotension (systolic arterial pressure less than 80 mmHg) with signs of left ventricular dysfunction, right ventricular infarction, and hypoperfusion due to mechanical complications lasting more than 30 minutes.

The patients were also assessed based on the Killip classification. Advanced heart failure is defined as a New York Heart Association (NYHA) classification of ≥ 3. DM involves the use of oral hypoglycemic agents or insulin therapy. Hypercholesterolemia was defined as a total cholesterol level of ≥ 200 mg/dL or higher.

Acute stent thrombosis is the occurrence of sudden cardiac symptoms (similar to ACS) within 24 hours after stent placement, accompanied by increased biomarker levels or electrocardiographic evidence of myocardial injury and angiographic evidence of thrombus obstructing flow near the previously implanted stent.

Cardiovascular mortality is sudden death of unknown cause, death due to AMI, heart failure, or arrhythmia. Recurrent infarction is an increase in serum CK-MB enzyme levels above twice the upper limit of normal and ST-segment re-elevation. Major adverse cardiac events (MACE) are cardiovascular mortality, recurrent infarction, and target vessel revascularization (TVR) (percutaneous or surgical). Only cardiovascular mortality is recorded.

Serious ventricular arrhythmias (ventricular tachycardia and/or fibrillation), stroke, cardiopulmonary resuscitation, advanced heart failure, atrioventricular block, temporary pacing intervention, intra-aortic balloon pump, atrial fibrillation, major bleeding requiring two or more units of blood transfusion, dialysis, acute stent thrombosis, and MACE are recorded during hospitalization.

Statistical analysis and interpretation

Patients are classified as having high or low MPV based on the upper limit of the normal range using a percentage value. Quantitative variables are expressed as mean ± SD, and qualitative variables are expressed as percentages (%). A two-tailed Student’s t-test is used to compare parametric values between the two groups. The chi-square test, or Fisher’s exact test, is used to compare categorical variables. Backward stepwise multivariate Cox regression analysis, including variables with p<0.1, is performed to identify independent predictors of cardiovascular mortality. Gender, age, reperfusion time longer than six hours, DM, hypertension, smoking habits, GFR lower than 60 mL/min/1.73 m^2^, multivessel disease, failed procedure, anterior MI, anemia at admission, blood transfusion, Killip higher than 1, and RDW higher than 14.8 are included in the model. The Kaplan-Meier analysis is used to generate cumulative survival curves for cardiovascular mortality, and the log-rank test is used to evaluate differences. A p-value of less than 0.05 is considered statistically significant. All statistical analyses were conducted using SPSS software (version 15.0, IBM Corp., Armonk, NY, USA).

## Results

Patient characteristics

The demographic, physical examination, angiographic, and baseline laboratory characteristics of the entire patient group are provided in Table 1.

**Table 1:** Clinical characteristics and laboratory findings of study participants. Killip: Killip classification; TIMI: thrombolysis in myocardial infarction; LDL: low-density lipoprotein; HDL: high-density lipoprotein; MPV: mean platelet volume.

Variable	Value (mean ± SD)
Age (year)	56.20 ± 12.90
Male, n(%)	356 (89)
Female, n(%)	44 (11)
Body mass index	26.60 ± 4.10
History	
Hypertension	113 (28)
Diabetes mellitus, n(%)	40 (10)
Hyperlipidemia, n(%)	70 (17)
Family history, n(%)	110 (27)
Smoking, n(%)	317 (79)
Previous myocardial infarction	40 (10)
Presentation findings	
Systolic blood pressure (mmHg)	122.10 ± 23.70
Diastolic blood pressure (mmHg)	71.90 ± 14.60
Pulse	78.50 ± 14.20
Pain duration (min)	74.10 ± 100.30
Killip class >1, n(%)	31 (8)
Anterior myocardial infarction, n(%)	198 (49)
Door-to-balloon time	32.90 ± 10.90
Those achieving TIMI 3 flow, n(%)	369 (92)
Left ventricular ejection fraction	45.50 ± 8.50
Laboratory findings	
Fasting serum glucose (mg/dL)	148.50 ± 73.60
Total cholesterol (mg/dL)	188.40 ± 62.50
LDL cholesterol (mg/dL)	118.10 ± 37.20
HDL cholesterol (mg/dL)	40.90 ± 11.40
Triglycerides (mg/dL)	137.10 ± 88.20
Creatinine (mg/dL)	0.90 ± 0.32
Creatine kinase-MB (U/L)	193.90 ± 172.90
Hemoglobin (g/dL)	14.20 ± 2.10
Hematocrit	40.40 ± 5.10
Leukocyte	13.40 ± 12.10
Platelet (10^3^/µL)	247.10 ± 73.60
MPV	8.67 ± 3.51

The mean patient age was 56.20 years, with a standard deviation of 12.90 years. The cohort comprised 356 males (89%) and 44 females (11%). The average body mass index (BMI) was 26.60 ± 4.10. Common comorbidities included hypertension (n = 113, 28%), DM (n = 40, 10%), hyperlipidemia (n = 70, 17%), and a family history of CVDs (n = 110, 27%). A significant proportion of the patients reported a history of smoking (n = 317, 79%) and myocardial infarction (n = 40, 10%). Additionally, 31 patients (8%) were classified as having Killip class >1, and nearly half of the cohort presented with anterior myocardial infarction (n = 198, 49%). The median door-to-balloon time was 32.90 ± 10.90 minutes, and 369 patients (92%) achieved TIMI 3 flow. The mean LVEF was 45.50 ± 8.5%. In terms of laboratory findings, fasting serum glucose, total cholesterol, LDL cholesterol, HDL cholesterol, and triglyceride levels were measured at 148.50 ± 73.60 mg/dL, 188.40 ± 62.50 mg/dL, 118.10 ± 37.20 mg/dL, 40.90 ± 11.40 mg/dL, and 137.10 ± 88.20 mg/dL, respectively. Creatinine, creatine kinase-MB, hemoglobin, hematocrit, leukocyte count, platelet count, and MPV were also assessed, yielding values of 0.90 ± 0.32 mg/dL, 193.90 ± 172.90 U/L, 14.20 ± 2.10 g/dL, 40.40 ± 5.1%, 13.40 ± 12.1 × 10^3^/µL, 247.1 ± 73.6 × 10^3^ /µL, and 8.67 ± 3.51 fL, respectively.

In-hospital outcomes

In-hospital adverse outcomes following primary PCI are presented in Table 2. MACEs are considered recurrent MI, TVR, and death.

**Table 2:** In-hospital adverse outcomes following primary PCI. MI: myocardial infarction; MACE: Major adverse cardiac events.

Outcome	Frequency
Recurrent MI, n(%)	16 (14)
Target vessel revascularization, n(%)	15 (3.8)
Severe ventricular arrhythmia, n(%)	45 (11.2)
Cardiogenic shock, n(%)	27 (6.8)
Death, n(%)	18 (4.5)
MACE, n(%)	35 (8.8)

Among the recorded events, recurrent myocardial infarction (MI) occurred in 16 patients (14%), whereas TVR was necessary in 15 patients (3.8%). Severe ventricular arrhythmia affected 45 patients (11.2%), and cardiogenic shock was observed in 27 patients (6.8%). Eighteen patients (4.5%) died during their hospital stay. MACEs occurred in 35 patients (8.8%).

When compared to survivors, deceased patients were found to be older, predominantly male, and more hypertensive, with a higher prevalence of diabetes, lower LVEFs, lower LDL levels, and lower levels of Hb and hematocrit. However, statistically significant differences were not found between the MPV values in both groups. A comparison of hospital follow-up outcomes between deceased patients and surviving patients is presented in Table 3.

**Table 3:** Comparison of hospital follow-up outcomes between deceased and surviving patients. MI: myocardial infarction; EF: ejection fraction; PCI: primary cutaneous intervention; TIMI: thrombolysis in myocardial infarction; CK-MB: creatine kinase-myoglobin binding; LDL: low-density lipoprotein; HDL: high-density lipoprotein; MPV: mean platelet volume. Values are presented as mean (standard deviation) for continuous variables and count (%) for categorical variables. P-values less than 0.05 are considered statistically significant.

Variable	Deceased patients (n=18, 4.5%)	Surviving patients (n=382, 95.5%)	P-value
Age (years)	66.90 (14.10)	55.70 (12.60)	<0.01
Male Gender (%)	11 (61.10)	345 (90.30)	<0.01
Smoking (%)	11 (61.10)	306 (80.10)	0.06
Hypertension (%)	11 (61.10)	102 (26.70)	<0.01
Diabetes Mellitus (%)	7 (38.90)	32 (8.40)	<0.01
Previous MI (%)	2 (11.10)	37 (9.70)	0.84
Anterior MI (%)	8 (44.40)	190 (49.70)	0.66
Left Ventricular EF (%)	34.10 (9.90)	46.10 (8.10)	<0.01
Post-PCI TIMI Flow <3 (%)	3 (16.70)	5 (1.30)	<0.01
Admission Glucose (mg/dL)	286.90 (141.50)	141.90 (61.80)	<0.01
Creatinine Level (mg/dL)	1.53 (0.76)	0.87 (0.25)	<0.01
Peak CK-MB (U/L)	445.90 (351.30)	181.90 (150.50)	<0.01
Triglycerides (mg/dL)	139.60 (81.20)	136.90 (88.70)	0.90
Total cholesterol (mg/dL)	159.60 (47.80)	189.90 (62.90)	0.04
LDL cholesterol (1-SD Increase)	85.80 (31.50)	119.50 (36.60)	<0.01
HDL cholesterol (mg/dL)	42.20 (14.50)	40.80 (11.30)	0.61
Hemoglobin (g/dL)	13.10 (1.80)	14.20 (2.10)	0.02
Hematocrit (%)	37.10 (4.90)	40.60 (4.90)	<0.01
Platelet (10^3^/µL)	244.60 (41.90)	247.30 (74.80)	0.88
MPV (fL)	8.60 (0.90)	8.67 (3.58)	0.93

Age, male gender, smoking, DM, hypertension, anterior MI, previous MI history, left ventricular EF, post-percutaneous intervention TIMI flow less than 3, creatinine level, hematocrit, LDL level, and MPV were subjected to univariate analysis, and those with p-values below 0.1 among these parameters were evaluated in multivariate analysis. Since MPV did not yield significant results in univariate analysis, it was not included in multivariate analysis. In multivariate analysis, diabetes, creatinine levels, and LDL levels showed significant results. Predictors of in-hospital MACEs are presented in Table 4.

**Table 4:** Predictors of in-hospital major adverse cardiac events. MI: myocardial infarction; EF: ejection fraction; TIMI: thrombolysis in myocardial infarction; LDL: low-density lipoprotein; MPV: mean platelet volume. Values are presented as odds ratios, along with their corresponding 95% confidence intervals (CI) in parentheses. Statistical significance was determined using p-values, with values less than 0.05 considered statistically significant.

Variables	Univariate analysis, odds ratio (95% CI)	P-value	Multivariate analysis, odds ratio (95% CI)	P-value
Age	1.04 (1.01-1.07)	0.004	0.99 (0.96-1.03)	0.86
Male gender	0.36 (0.15-0.87)	0.023	0.39 (0.12-1.27)	0.12
Smoking	0.87 (0.38-1.98)	0.748	-	-
Diabetes mellitus	5.55 (2.45-12.44)	<0.01	3.45 (1.24-9.63)	0.02
Hypertension	4.31 (1.62-11.40)	<0.01	1.48 (0.59-3.70)	0.40
Previous myocardial infarction	1.62 (0.59-4.45)	0.345	-	-
Anterior MI	0.57 (0.28-1.17)	0.131	-	-
Left ventricular EF, 1-SD decrease	1.08 (1.04-1.12)	<0.01	1.02 (0.97-1.07)	0.27
Post-PCI TIMI flow <3	3.38 (1.26-9.05)	0.015	3.51 (0.57-21.40)	0.17
Creatinine level, 1-SD increase	12.30 (4.43-34.12)	<0.01	6.46 (2.17-19.20)	<0.01
Hematocrit, 1-SD decrease	1.08 (1.02-1.15)	<0.01	0.98 (0.90-1.06)	0.62
LDL cholesterol, 1-SD decrease	1.02 (1.01-1.03)	<0.01	1.02 (1.01-1.03)	0.03
MPV, 1-SD increase	0.98 (0.84-1.15)	0.83	-	-

**Figure 1:**
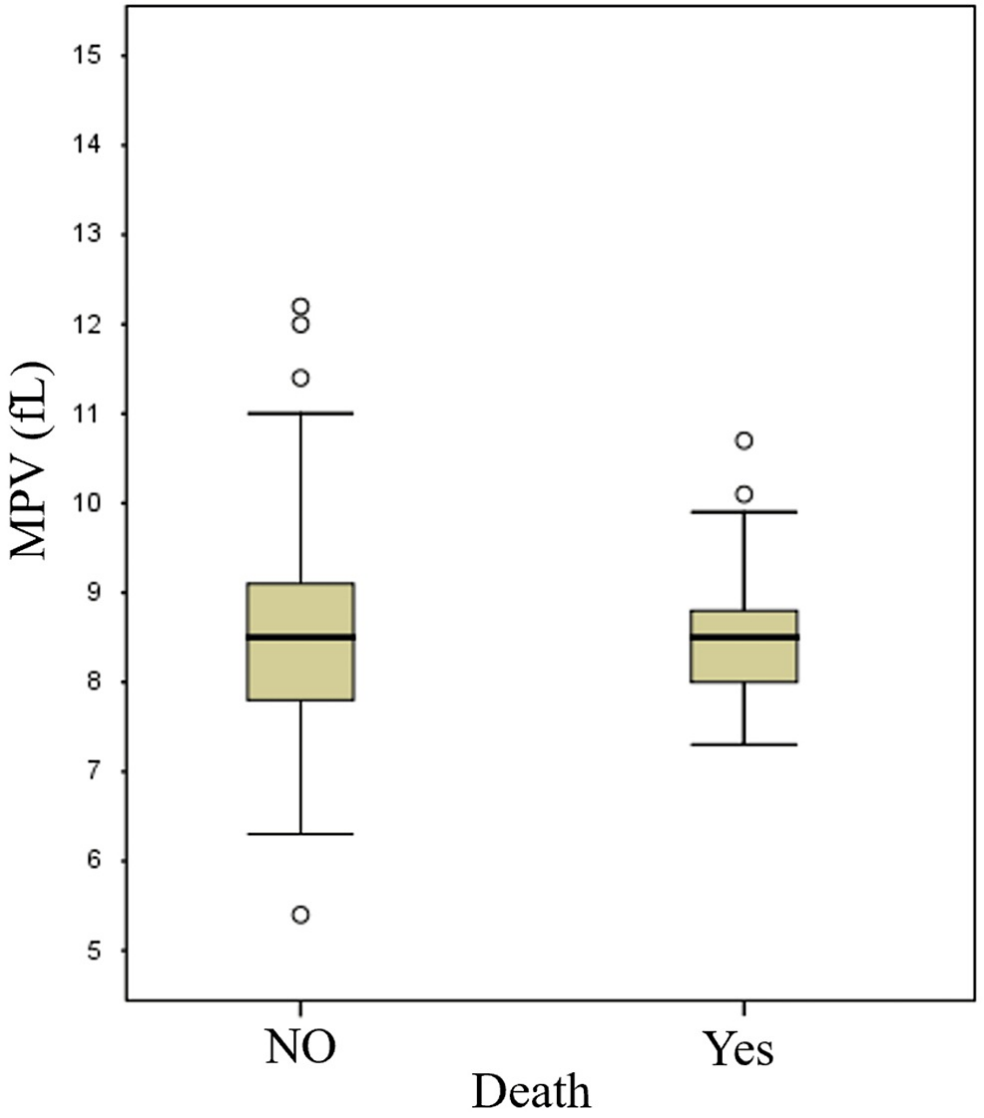
Comparison of MPV values between in-hospital deceased and surviving patients. MPV: mean platelet volume.

A comparison of the MPV values between in-hospital deceased and surviving patients is shown in Figure 1.

**Figure 2:**
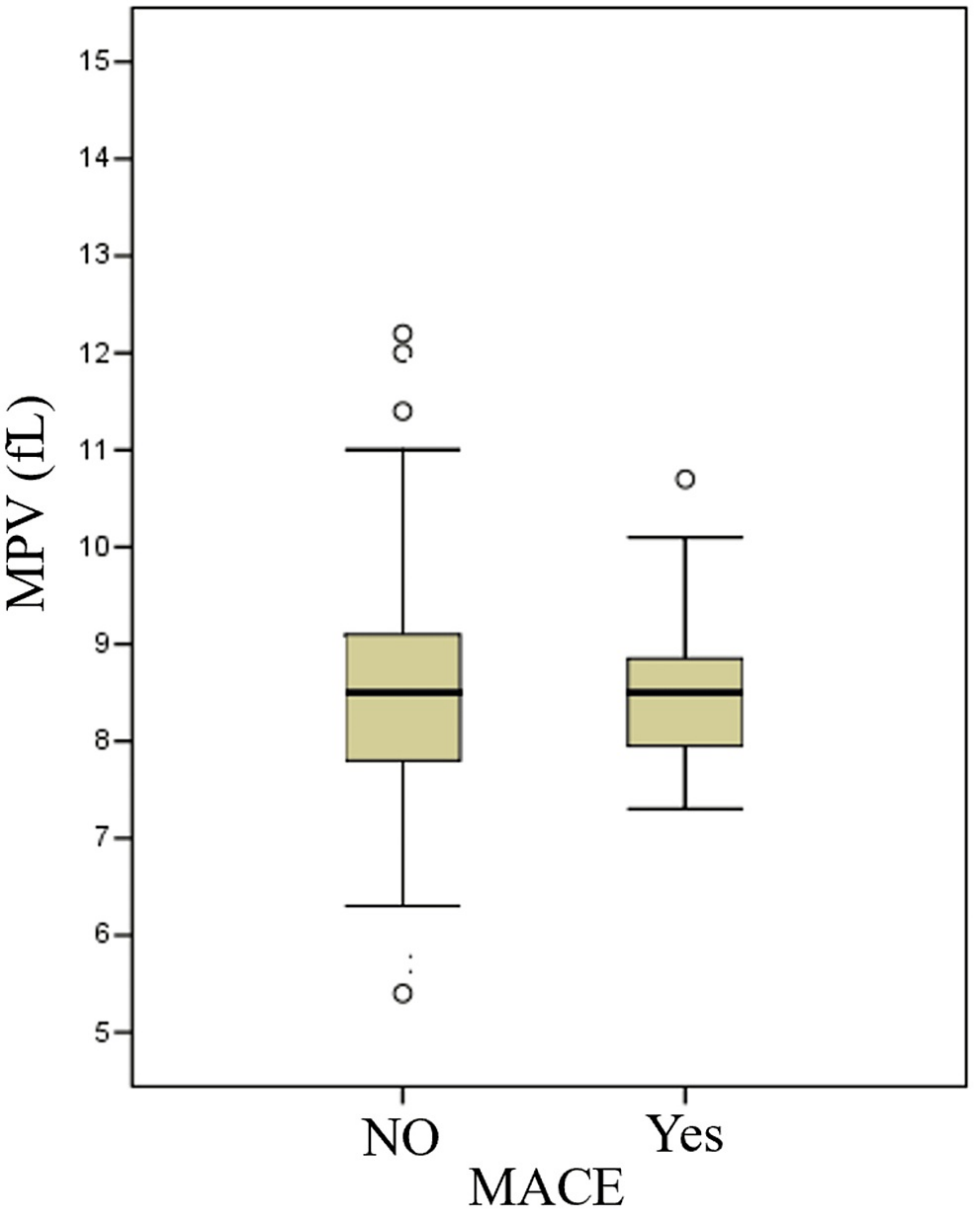
Illustration of MPV values in patients developing in-hospital MACE. MPV: mean platelet volume; MACE: Major adverse cardiac events.

The MPV values in patients with in-hospital MACE are illustrated in Figure 2.

## Discussion

This study examined the impact of MPV on in-hospital death and complications in patients who received PCI for STEMI. We found no significant difference in MPV levels between deceased patients and survivors. We also did not find any significant association between MPV levels and adverse in-hospital outcomes, such as repeat MI, TVR, and events categorized as mortality.

Ex vivo platelet function measurement is an effective way to show platelet function [14]. Platelets in circulation have different sizes, densities, and activities [15]. It is thought that this heterogeneity may be one of the factors causing ACS [16]. Following plaque rupture within the coronary artery, activated large and proaggregatory platelets significantly contribute to thrombus formation [17]. Platelet sizes are used as a marker of platelet activity. MPV may also indicate platelet activation or physiology, as well as platelet production speed or biology [18]. Larger platelets are more metabolically and enzymatically active than smaller ones. They have more substances that promote blood clotting, have higher levels of thromboxane A2 and B2, and display more glycoprotein IIb-IIIa receptors. Activated platelets clump together more strongly in response to adenosine diphosphate (ADP) and are less inhibited by prostacyclins in vitro. Large platelets have more and bigger α-granules that contain substances that promote blood clotting, such as platelet factor 4, P-selectin, and platelet-derived growth factor. They also produce factors that attract and stimulate other cells, which further increase neointimal growth. Finally, large platelets are linked to a higher number of immature red blood cells as they are newly released from the bone marrow. This phenomenon is an independent predictor of reduced response even in dual antiplatelet therapies [19-24].

The process of blood clotting that is triggered by a break or tear of an unstable plaque in the artery wall causes clinical events in ACSs. Considering that MPV, an easily accessible marker reflecting platelet functions, which play one of the most active roles in the formation and growth of intracoronary thrombus, could be utilized as a supportive diagnostic marker in ACSs, we hypothesized that MPV values at the time of hospital admission may have prognostic value for in-hospital mortality and morbidity in these patients.

In our study, MPV levels at admission were not related to in-hospital events or death, but Huczek et al. found a link between MPV and reduced blood flow in the artery after primary percutaneous intervention and an even higher death rate at six months [25]. Likewise, Estévez-Loureiro et al. found a significant association between high MPV and 30-day in-hospital death among patients with TIMI 2 or 3 flow [26]. Martin et al. showed that MPV, measured after six months in patients with acute myocardial infarction, was an independent risk factor for another myocardial infarction [27]. Moreover, Martin et al., in a study comparing patients with myocardial infarction and healthy controls, saw that the difference in MPV levels lasted more than six weeks [28].

Our study results differ from the findings of previous studies, which had long-term follow-ups with patients. We focused on patients with STEMI who underwent primary percutaneous intervention instead of covering all ACSs. We did not compare admission MPV values with a normal control group directly. However, many previous studies have shown that admission MPV values in patients with ACS are significantly higher than in healthy individuals [29]. This observation may also apply to our patient group, although it is not certain.

However, the absence of a link between higher admission MPV values and major cardiac events in the hospital, including death, in our study may be due to the fact that all these patients had already started atherosclerotic processes. Other factors may become important once this process begins. We performed univariate analysis with age, male gender, smoking, DM, hypertension, anterior myocardial infarction, history of previous myocardial infarction, LVEF, presence of less than TIMI 3 flow after percutaneous intervention, creatinine level, hematocrit, and LDL level to examine which parameters influence in-hospital events, and then found diabetes, creatinine level, and LDL level as significant factors in multivariate analysis. However, it is possible that the continuous increase in MPV, even after treatment and discharge, may indicate a continuous risk of atherosclerotic thrombosis in patients.

The number of patients in our study is large enough compared to other studies. However, our study has limitations that need consideration. Its retrospective design may introduce biases, and the focus solely on STEMI patients undergoing PCI limits generalizability. Only assessing in-hospital outcomes overlooks longer-term effects post-discharge. The MPV values at admission were not compared with a normal control group directly. Despite attempts to adjust for confounders, unmeasured factors may influence results. Routine MPV measurement introduces variability, and being a single-center study reduces external validity. Additionally, the disproportionate male-to-female ratio observed in our study limits generalizability of our findings.

## Conclusions

In conclusion, this study found that MPV levels upon admission were not predictive of in-hospital outcomes in patients with STEMI undergoing primary PCI. Despite previous research suggesting a potential association between elevated MPV and adverse cardiovascular events, our findings did not support this relationship within the context of our study population and timeframe. Future prospective multicenter studies with longer follow-up periods and standardized protocols are needed to address the limitations of our study and enhance understanding of MPV's prognostic value in STEMI patients undergoing PCI.
